# Prospective Control in Catching: The Persistent Angle-of-Approach Effect in Lateral Interception

**DOI:** 10.1371/journal.pone.0080827

**Published:** 2013-11-22

**Authors:** Simon Ledouit, Remy Casanova, Frank T. J. M. Zaal, Reinoud J. Bootsma

**Affiliations:** 1 Institut des Sciences du Mouvement, Aix-Marseille Université, CNRS, Marseille, France; 2 Center for Human Movement Sciences, University Medical Center Groningen, University of Groningen University, The Netherlands; University of California, Merced, United States of America

## Abstract

In lateral interception tasks balls converging onto the same interception location via different trajectories give rise to systematic differences in the kinematics of hand movement. While it is generally accepted that this angle-of-approach effect reflects the prospective (on-line) control of movement, controversy exists with respect to the information used to guide the hand to the future interception location. Based on the pattern of errors observed in a task requiring visual extrapolation of line segments to their intersection with a second line, angle-of-approach effects in lateral interception have been argued to result from perceptual biases in the detection of information about the ball's future passing distance along the axis of hand movement. Here we demonstrate that this account does not hold under experimental scrutiny: The angle-of-approach effect still emerged when participants intercepted balls moving along trajectories characterized by a zero perceptual bias with respect to the ball's future arrival position (Experiment 4). Designing and validating such bias-controlled trajectories were done using the line-intersection extrapolation task (Experiments 2 and 3). The experimental set-up used in the present series of experiments was first validated for the lateral interception and the line-intersection extrapolation tasks: In Experiment 1 we used rectilinear ball trajectories to replicate the angle-of-approach effect in lateral interception of virtual balls. Using line segments extracted from these rectilinear ball trajectories, in Experiment 2 we replicated the reported pattern of errors in the estimated locus of intersection with the axis of hand movement. We used these errors to develop a set of bias-free trajectories. Experiment 3 confirmed that the perceptual biases had been corrected for successfully. We discuss the implications on the information-based regulation of hand movement of our finding that the angle-of-approach effect in lateral interception cannot not explained by perceptual biases in information about the ball's future passing distance.

## Introduction

Success in interceptive actions requires getting to the right place at the right time [1]. Although the organization of interception movements may be based on accurate perceptual estimates of the future place and time of contact, a large body of work provides convincing evidence for a more robust alternative, based on a continuous, functional coupling between information and movement. Here we will refer to the former type of organization as *predictive control* and to the latter as *prospective control* [1]–[4]. Consensus has emerged over the last two decades limiting the operation of predictive control to explosive movements of short duration [5]–[11]. For interceptive movements of sufficiently long duration, actions are characterized by the pursuit of particular states of the agent-environment interaction that guarantee (i.e., are lawfully related to) the future achievement of the goal. Thus, in a prospective control scheme the unfolding movement is based on time-evolving information with respect to what the agent must do so as to ensure interception, without requiring precise knowledge of when and where this will occur. While prospective strategies have been documented for locomotor (whole-body displacement) interception tasks, both in humans [12]–[19] and animals [20]–[23], here we concentrate on manual (lateral) interception.

**Figure 1:**
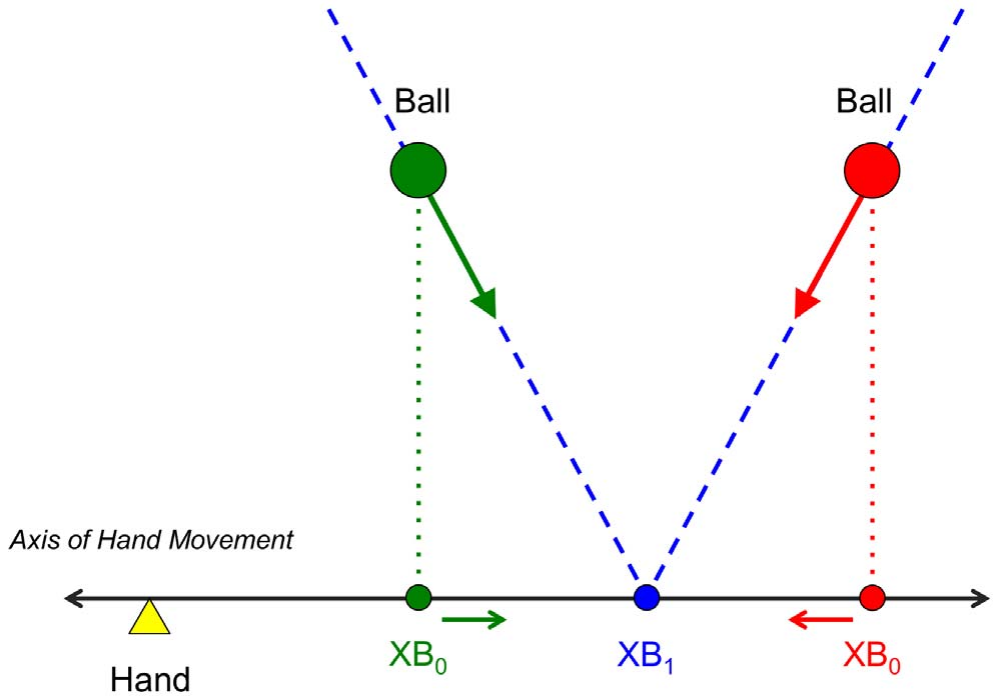
Definition of variables. XB_0_ is the current lateral position of the ball projected orthogonally on the interception axis (axis of hand movement). XB_1_ is the future lateral position of the ball on the interception axis if current heading is maintained. Balls (green and red circles) moving along rectilinear trajectories (dashed blue lines) with constant velocity (fat green and red arrows) will cross the axis of hand movement at position XB_1_ (blue circle). For balls moving in the observer's transverse plane [1], [24]–[28] with the point of observation located on the axis of hand movement, optical specification is defined in angles of ball eccentricity (*θ*) and ball size (*φ*) In this case, XB_0_ and XB_1_ are optically specified, in units of ball size, by sin*θ*/tan*φ* and 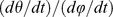, respectively [27], [36], [37]. For balls moving in a plane perpendicular to the observer's line of sight (present study) with the point of observation located at an orthogonal distance *D* from the axis of hand movement, optical specification is defined in angles of ball azimuth (*α*) and ball elevation (*ε*). For small angles, distances XB_0_ and XB_1_ are optically specified, in units of distance *D*, by *α* and 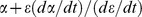, respectively.

Peper et al. [1] were the first to demonstrate systematic differences in the kinematic patterns of hand movement when participants caught balls following different trajectories converging onto the same interception location and arriving there after the same flight duration. This *angle-of-approach effect* is incompatible with a movement control strategy based on accurately predicted place and time of contact because these were invariant over the different trajectories. Although the influence of the ball's motion trajectory on the kinematics of interception movements has been replicated on several occasions [24]–[30], the nature of the information underlying the prospective control of lateral interception is still subject of debate. While differing in dynamical structure, all existing models of prospective control of lateral interception [1], [2], [25], [27], [30], [31] are based on the idea that the hand is continuously attracted toward an informationally-specified, time-evolving position along the interception axis. A first controversy exists as to whether this hand-attractor position is based on the projection of the current lateral position of the ball onto the interception axis [1], [2], [25], [31] (informationally-specified zero-order variable XB_0_, see Fig. 1) or on the lateral position where the ball will cross the interception axis if its current direction of motion is maintained [27], [28], [30] (informationally-specified first-order variable XB_1_, see Fig. 1).

As can be seen from Fig. 1, for a rectilinear ball trajectory the ball's current projected position XB_0_ continuously varies during the approach to the interception point. Moreover for different rectilinear ball trajectories converging onto the same position on the interception axis, XB_0_ evolves in a different way. On the other hand, the future passing position XB_1_ is the same and remains invariant throughout the approach for all rectilinear ball trajectories converging onto the same interception position. Thus, the kinematic patterns of hand movement produced when participants intercept balls following different rectilinear trajectories converging onto the same interception position was hypothesized to allow experimental discrimination between these two candidate information sources [1], [2]. Finding an angle-of-approach effect under these conditions would challenge accounts based on the use of XB_1_-based information.

In a study using rectilinear ball trajectories, Montagne et al. [24] did find a systematic angle-of-approach effect on the kinematics of lateral catching movements. This finding led them to reject the use of XB_1_-based information and to conclude in favour of the use of XB_0_-based information. However, Arzamarski et al. [28] recently questioned this interpretation, thereby initiating a second controversy. They suggested that participants would in fact use XB_1_-based information but that perceptual biases herein were responsible for the angle-of-approach effect. To provide evidence for the existence of such biases in perceived future ball crossing position, they examined participants' performance on a line-intersection extrapolation task. In this task, line segments (conceived as static representations of segments of rectilinear ball trajectories) were to be extrapolated to the intersection with a second line (corresponding to the axis of hand movement). Participants' estimates of the intersection position revealed systematic errors across line-segment orientations to and distances from the axis of hand movement. These perceptual biases identified in the static line-intersection extrapolation task were interpreted as providing evidence in favour of perceptual biases in the detection of XB_1_-based information in the dynamic interception task [28].

In the present contribution we experimentally tested whether perceptual biases observed in a line-intersection extrapolation task can really explain the angle-of-approach effects observed in lateral interception. To this end, we set out to experimentally construct a set of ball-motion trajectories for which the perceptual bias with respect to the ball's future arrival position was controlled to be effectively zero at each point of each trajectory. If a systematic angle-of-approach effect were still to be observed when intercepting balls moving along these bias-controlled trajectories, this would disqualify the perceptual bias explanation proposed by Arzamarski et al. [28]. As a consequence, the existing body of results would not be compatible with the exclusive use of XB_1_-related information in the prospective control of lateral interception [27], [28], [30].

Because we used a new experimental interception setup with virtual balls moving in a plane perpendicular to the participant's line of sight, we proceeded in four steps. In Experiment 1 we sought to replicate the angle-of-approach effect on interception movements, generally observed for balls moving in the participants' transverse plane [1], [24]–[28]. Rectilinear ball trajectories converging onto the same interception locations gave rise to reliably different, trajectory-dependent patterns of interceptive hand movement. The angle-of-approach effects observed in Experiment 1 were equivalent to those reported in the literature, thereby validating our new experimental setup for the interception task. In Experiment 2 we sought to replicate the perceptual biases reported by Arzamarski et al. [28]. We had participants perform the line-intersection extrapolation task used by Arzamarski et al. [28] in our new experimental setup. To this end, we replaced the moving balls of Experiment 1 with static line segments, corresponding to segments of the rectilinear ball trajectories that these balls had followed. Participants' estimations of the intersection locus of these line segments with the axis of hand movement revealed systematic errors: Biases varied with the orientation of the line segments to and their distance from the axis of hand movement. The pattern of result was equivalent to that reported by Arzamarski et al. [28], thereby also validating our experimental setup for the line-intersection extrapolation task.

In Experiment 3 we tested whether the systematic nature of biases identified in Experiment 2 could be used to control participants' estimates of the intersection locus in the line-intersection extrapolation task. Based on the relation of the biases observed with segment orientation and distance, we generated a new set of (slightly curved) trajectories that where all characterized by a predicted zero-bias with respect to the future arrival position, at each point in the trajectory. Estimates of the intersection locus for line segments derived from these new trajectories no longer revealed systematic errors, demonstrating that it was indeed possible to control for bias in the line-intersection extrapolation task. Finally, in Experiment 4, we had participant intercept balls moving along these bias-controlled trajectories. The angle-of-approach effect on the kinematics of interception movements observed Experiment 1 for rectilinear ball trajectories still emerged when participants intercepted balls moving along the bias-controlled trajectories. The present series of experiments thereby provides compelling evidence against the perceptual bias explanation for angle-of-approach effects in lateral interception.

## Experiment 1: Intercepting balls moving along rectilinear trajectories

The goal of this first experiment was to validate a new experimental setup for lateral interception of virtual balls moving in a plane perpendicular to the participant's line of sight. To this end we sought to replicate the angle-of-approach effect on interception movements, generally observed for balls moving in the participants' transverse plane [1], [24]–[28].

### Ethics Statement

For this, as for the subsequent experiments reported in the present contribution participants provided written consent prior to participation. The study was approved by the local institutional review board (IRB) of the Institute of Movement Sciences (*Comité Ethique de l'Institut des Sciences du Mouvement d'Aix-Marseille Université*) and conducted according to University regulations and the Declaration of Helsinki.

### Materials and Methods

#### Participants

Five right-handed participants (2 men and 3 women, mean age 26.6±5.5 yrs) voluntarily took part in the experiment.

#### Task and Procedure

**Figure 2:**
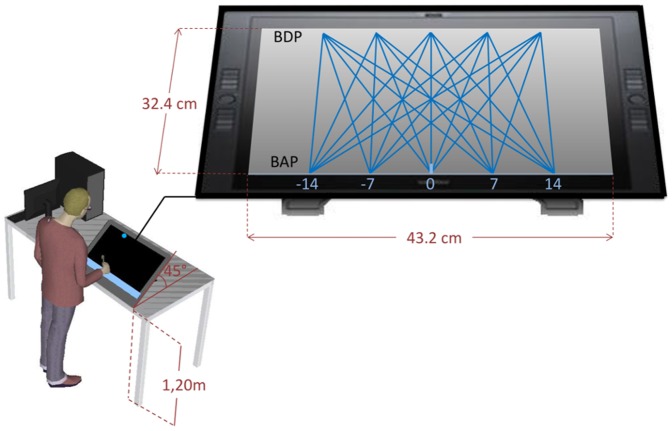
Representation of the experimental set-up. Starting from a fixed initial position (represented here by a vertical light blue line segment positioned at BAP = 0 cm) participants moved the hand-held stylus along the (horizontal) interception axis to intercept virtual balls moving from one of five Ball Departure Positions (BDP) to one of five Ball Arrival Positions (BAP).

The experiment took place in a darkened room without windows. The participant stood in front of an interactive Cintiq 21UX Wacom® tablet (screen size 43.2×32.4 cm, 1600×1200 pixel resolution) positioned at a height of 1.20 m and oriented at a 45° angle, providing a plane of motion perpendicular to the participant's line of sight (see Fig. 2). The task was to intercept simulated balls moving downward (top-to-bottom) across the tablet's screen by laterally displacing a hand-held stylus. To this end, participants moved the stylus along the top edge of a transparent, 5-cm wide plastic ruler, horizontally fixed to the tablet at the level of the bottom of the screen.

The interception axis was represented on the screen by a horizontal, 0.05-cm wide, blue line. Stylus position, sampled at a frequency of 100 Hz, was indicated by a vertical, 0.1-cm wide, white line cursor centred on the interception axis.

Before the onset of a trial the participant positioned the stylus cursor on the designated starting point, located at the centre of the interception axis. This point was used to define the X-Y origin of the screen, X increasing negatively to the left and positively to the right of the starting position and Y increasing positively to the top of the screen. When the stylus cursor was correctly positioned at the starting position, a ball, represented by a 0.8-cm diameter white circle against a black background, appeared at one of the five possible departure positions (Y = +32 cm; X = −14, −7, 0, +7, or +14 cm). After remaining stationary for 3 s, the ball moved at constant velocity across the screen towards one of five possible arrival positions along the interception axis (Y = 0 cm; X = −14, −7, 0, +7, or +14 cm). Combining the five departure positions and the five arrival positions gave rise to 25 different rectilinear ball trajectories. Balls could move at vertical (Y) speeds of 20 or 32 cm/s, for motion durations of 1.6 or 1.0 s. Participants performed 5 blocks of 50 trials, with the order of the 50 conditions (25 trajectories × 2 ball speeds) randomized over trials within each block. Feedback with respect to interception (yes/no) was automatically provided at the end of each trial, with successful interception requiring that the distance between stylus and ball was less than 0.45 cm (half the sum of ball diameter and cursor width).

During the experiment, ball and stylus positions were sampled at a frequency of 100 Hz and stored on disk for each individual trial. Before data analysis the stylus position time-series were filtered using a second-order Butterworth filter with a cut-off frequency of 5 Hz [1], [24], [28], [32].

#### Data analysis

Interception performance was assessed using constant error, defined as the distance between ball and stylus position at the moment the ball crossed the interception axis. General trends in movement kinematics were captured in ensemble averages of the time series of position and velocity of stylus displacement. In order to statistically test differences in movement kinematics, we analysed (i) the position of the stylus at 400 ms before the ball crossed the interception axis and (ii) peak velocity of the stylus movement. The effect of approach trajectory on the pattern of interceptive movement was visible almost immediately after the start of the movement. The same qualitative pattern of results was found for stylus position at time-to-contacts (TTC) of 600, 400, and 200 ms.

All dependent variables–Constant Error, Stylus Position at TTC = 400 ms, and Peak Velocity–were submitted to repeated-measures Analyses of Variance (ANOVA) with factors Ball Speed (2 levels), Ball Departure Position (5 levels), and Ball Arrival Position (5 levels). Where appropriate significant (*p*<.05) main effects and interactions were further analysed using Newman-Keuls post-hoc tests.

### Results

**Figure 3:**
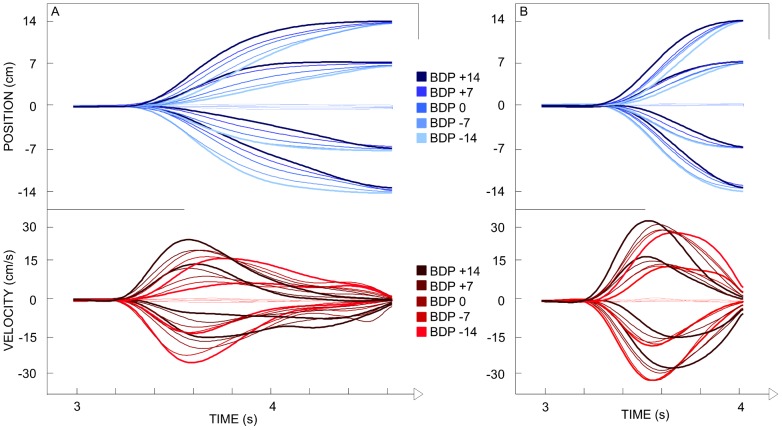
Ensemble averages of stylus position and velocity as a function of time (Exp. 1). The ball started moving 3(t_0_) on the screen. BDP: Ball Departure Position. Panel A: lower ball speed. Panel B: higher ball speed.

For each ball arrival position the five different ball departure positions corresponded to five different angles of approach (five different trajectories) to the same interception point. As can be seen from the ensemble averages of stylus position and velocity over time (Fig. 3), participants hardly moved the stylus when the ball would arrive at the stylus starting position (X = 0 cm). For all other ball arrival positions (X = −14, −7, +7, +14 cm) systematic effects of ball departure position (and hence angle of approach) were observed. These angle-of-approach effects were corroborated by the statistical analyses of kinematic characteristics of the movement patterns described below.

#### Performance

Interception performance was quite good, with 84.3% of the balls being intercepted. Overall, Constant Error was −0.02±0.57 cm. The ANOVA on Constant Error revealed significant main effects of Ball Speed (*F*(1, 4) = 177.56, *p*<.001), Ball Departure Position (*F*(4, 16) = 10.36, *p*<.001) and Ball Arrival Position (*F*(4, 16) = 13.78, *p*<.001). Inspection of the data revealed that the effects were mainly due to a larger (negative) Constant Error for the Ball Arrival Position  =  −14 cm (Ball Departure Positions  =  +14, +7, 0 cm) conditions with lower ball speed. As can be seen from Fig. 3, these effects were quite modest.

#### Movement kinematics

**Figure 4:**
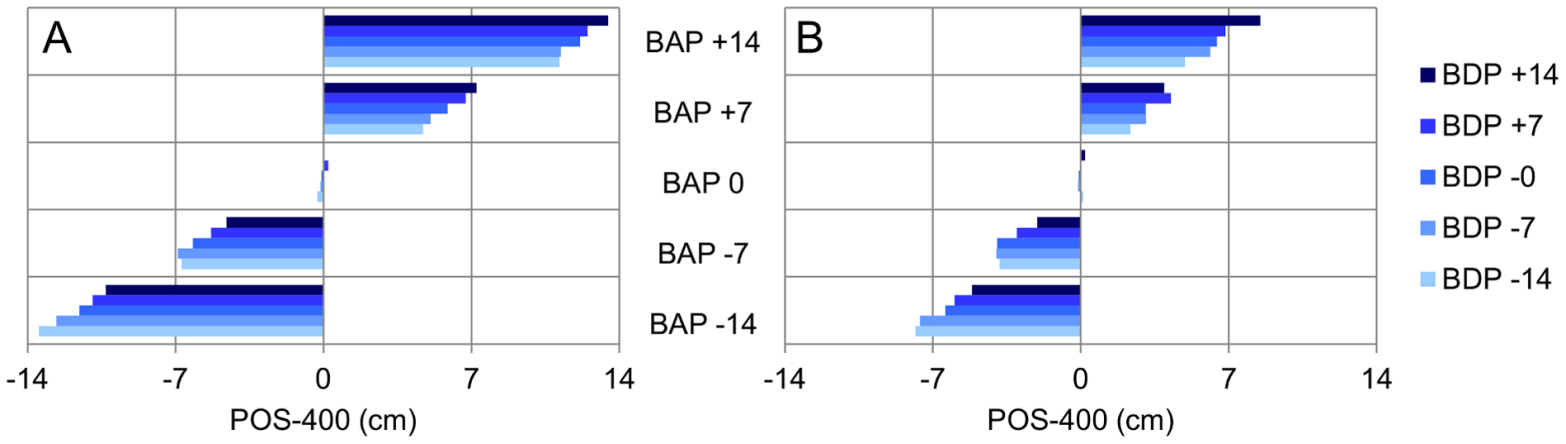
Pos-400 as a function of BDP and BAP (Exp. 1). Pos-400: Stylus position at 400 ms before the ball reached the interception axis. BDP: Ball Departure Position. BAP: Ball Arrival Position. Panel A: lower ball speed. Panel B: higher ball speed.

The ANOVA on the stylus position at TTC = 400 ms (Pos-400) revealed significant main effects of Ball Departure Position (*F*(4, 16) = 61.34, *p*<.001) and Ball Arrival Position (*F*(4, 16) = 1018.91, *p*<.001), as well as significant first-order interactions for Ball Speed × Ball Arrival Position (*F*(4, 16) = 183.96, *p*<.001) and Ball Departure Position × Ball Arrival Position (*F*(16, 64) = 6.18, *p*<.001). Post-hoc analysis of these effects brought out the following points (see Fig. 4). Overall, Pos-400 was further away from the starting position for (a) balls moving at the lower speed (all *p*s<.05) and (b) balls moving towards farther arrival positions (all *p*s<.05). The Ball Departure Position × Ball Arrival Position interaction indicated that for each arrival position Pos-400 varied systematically with the ball's angle of approach (at least two significant (*p*<.05) Ball Departure Position comparisons at each Ball Arrival Position, except for Ball Arrival Position  = 0 cm). The larger the (absolute) angle of approach, the further the stylus was from the future ball arrival position. The effect of angle of approach was observed for both ball speeds.

**Figure 5:**
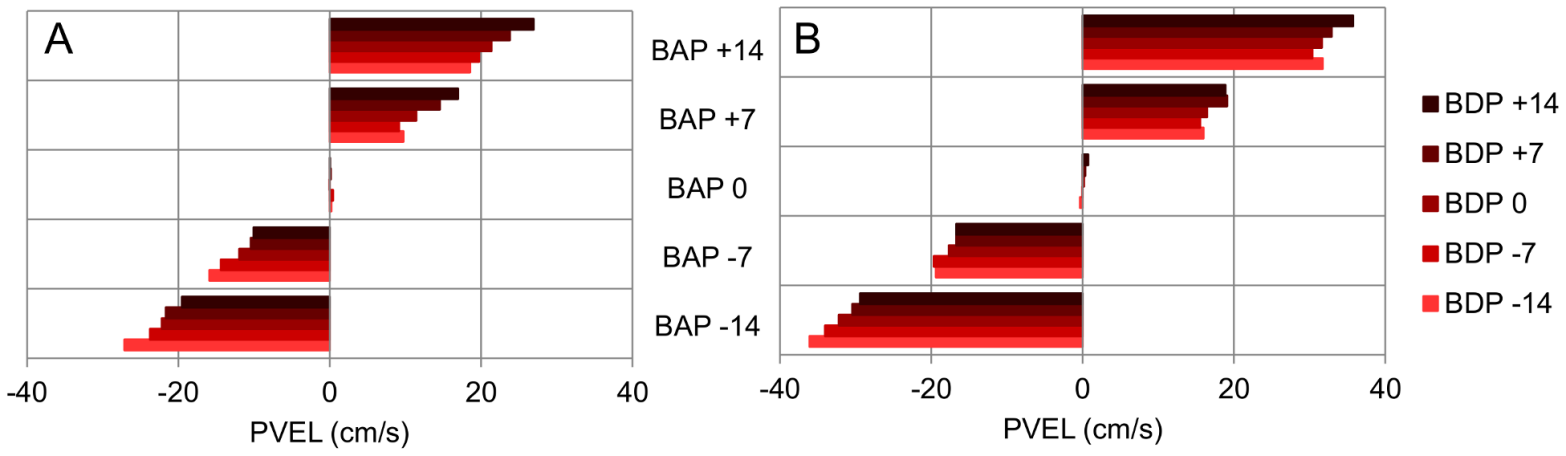
PVel as a function of BDP and BAP (Exp. 1). PVel: Peak Velocity. BDP: Ball Departure Position. BAP: Ball Arrival Position. Panel A: lower ball speed. Panel B: higher ball speed.

The ANOVA on Peak Velocity revealed significant main effects of Ball Departure Position (*F*(4, 16) = 34.57, *p*<.001) and Ball Arrival Position (*F*(4, 16) = 373.58, *p*<.001) as well as significant first-order interactions for Ball Speed × Ball Arrival Position (*F*(4, 16) = 182.19, *p*<.001) and Ball Departure Position × Ball Arrival Position (*F*(16, 64) = 2.85, *p*<.001). Post-hoc analysis of the interactions revealed several points (see Fig. 5). First, Peak Velocity was systematically larger when the distance between the initial stylus position and the Ball Arrival Position was larger (all *p*s<.05). Second, for each combination of Ball Departure Position and Ball Arrival Position, Peak Velocity was systematically larger when the ball moved faster (all *p*s<.05). Finally, for each Ball Arrival Position, Peak Velocity systematically varied with Ball Departure Position (at least one significant Ball Departure Position comparison at each Ball Arrival Position, except for Ball Arrival Position  = 0 cm), with smaller Peak Velocities being attained for ball trajectories with larger (absolute) angles of approach. This angle-of-approach effect was observed for both ball speeds.

### Discussion

The general characteristics of the movement patterns observed, such as higher peak velocities when larger distances were to be covered and higher peak velocities when balls moved faster, correspond to those reported in earlier studies of interception [1], [5], [24], [25], [27], [28], [33], [34]. Moreover, as in the earlier lateral interception studies [1], [24]–[28], systematic effects of the angle of approach of the ball's trajectory to the interception point were observed. Finally, the absence of hand movement when balls moved rectilinearly towards the initial hand position replicated the results reported by Arzamarski et al. [28]. Overall, these results thus validate our new experimental set-up for lateral interception of virtual balls moving in a plane perpendicular to the participant's line of sight.

## Experiment 2: Line-intersection extrapolation for rectilinear trajectories

Studying lateral interception of balls rolling–along rectilinear trajectories–across a table top, Arzamarski et al. [28] reported a pattern of results that, on all points, closely resembles the results of our Experiment 1. They argued that the observed angle-of-approach effect need not be interpreted as revealing the influence of information with respect to current lateral ball position (XB_0_). Rather, they suggested that this effect would stem from perceptual biases in establishing XB_1_. To demonstrate the existence of such perceptual biases in perceived future ball crossing position Arzamarski et al. [28] evaluated performance on a line-intersection extrapolation task. Using our new experimental setup, in Experiment 2 we adopted the same methodology in order to replicate their findings and to identify the characteristics of such biases.

### Materials and Methods

#### Participants

Ten right-handed participants (6 men and 4 women, mean age 21.7±1.7 yrs) voluntarily took part in the experiment. None of them had participated in Experiment 1.

#### Task and Procedure

The experimental set-up was the same as in Experiment 1, with the exception of one characteristic. Instead of laterally intercepting balls moving across the screen, participants were now asked to point to the position on the interception axis that corresponded to its intersection with a static line segment presented on the screen. Stylus position was recorded when participants marked the intersection position by pushing down the stylus. The line segments corresponded to parts of the 25 rectilinear trajectories used in Experiment 1. Four segments with a standardized 6-cm vertical (Y) extent were extracted from each trajectory, with the centre point of the segment corresponding to vertical (Y) distances from the interception axis of 3.0, 11.7, 20.3, or 29.0 cm. The segments centred on a vertical distance of 3.0 cm in fact touched the interception axis with their lowest points. They were used to ascertain that the pointing task itself did not introduce any supplementary biases.

A trial consisted of the presentation of a static line segment that remained visible until the participant marked the perceived intersection position. Participants performed 5 blocks of 100 trials, with the order of the 100 conditions (4 distances × 25 trajectories) randomized over trials in each block. No feedback was provided.

#### Data analysis

Performance of intersection locus estimation was assessed using Constant Error, defined as the distance between the required position (corresponding to the ball arrival position of the matching trajectory) and the marked stylus position. In order to allow a comparison with the results of Experiment 3 the line segments extracted from each of the 25 trajectories were characterized by their orientation with respect to the axis of hand movement. The 25 combinations of ball departure and arrival positions gave rise to 9 Segment Orientations (−41.2, −33.3, −23.6, −12.3, 0.0, 12.3, 23.6, 33.3, and 41.2°). Using a repeated-measures ANOVA, we evaluated the effects on Constant Error of the factors Segment Distance (4 levels) and Segment Orientation (9 levels).

### Results and Discussion

**Figure 6:**
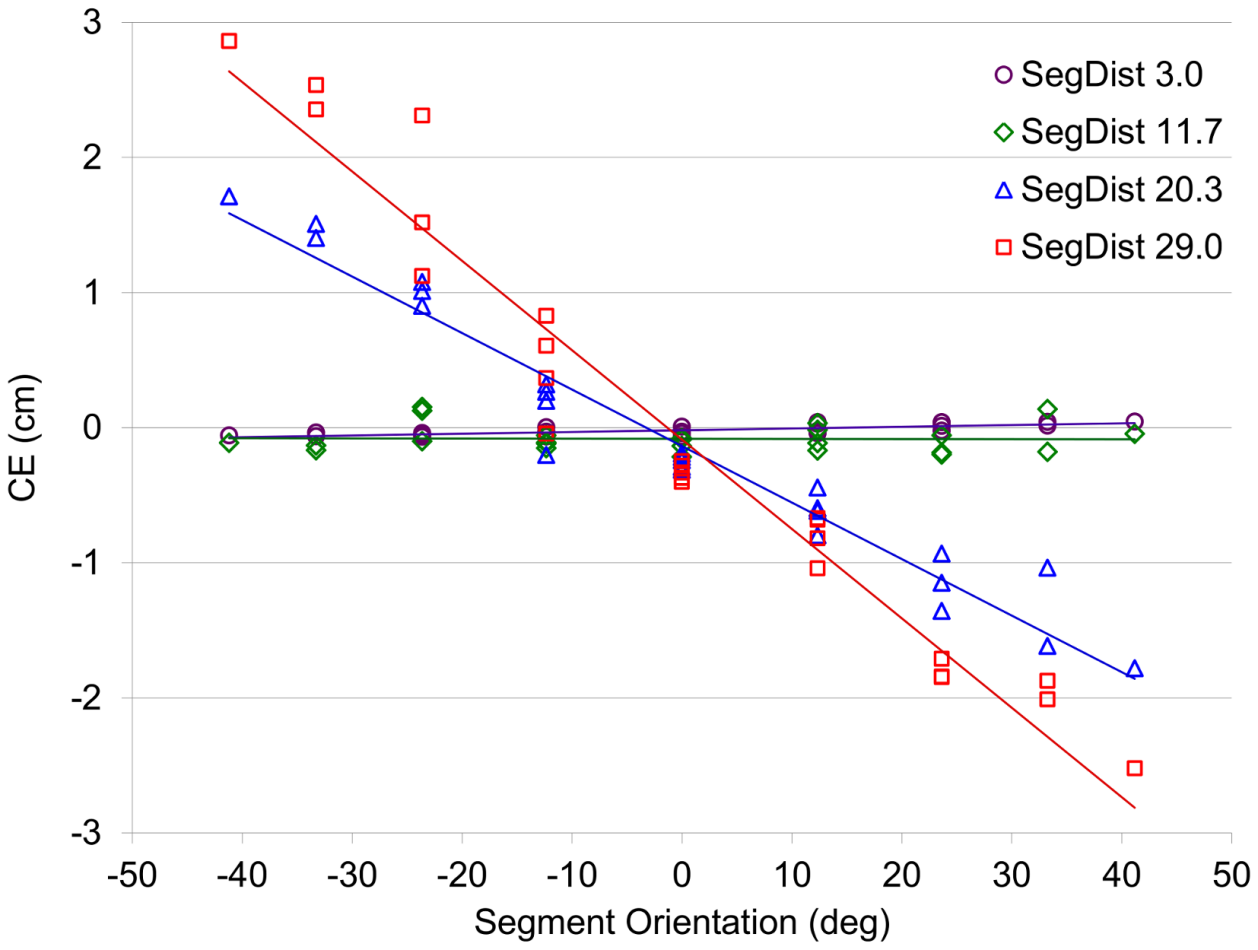
CE for intersection extrapolation as a function of segment orientation for the 4 segment distances (Exp. 2). CE: Constant Error. SegDist: Segment Distance.

The line-intersection extrapolation task revealed a systematic, distance-dependent influence of segment orientation on the perceived location of the intersection with the axis of hand movement (Fig. 6). Perceptual biases were analysed using the errors in intersection location estimation induced by segment orientation at different distances from the axis of hand movement.

The ANOVA on Constant Error in estimated intersection locus revealed a significant main effect of Segment Orientation (*F*(8, 72) = 11.59, *p*<.001) and a Segment Orientation × Segment Distance interaction (*F*(24, 216) = 17.82, *p*<.001). As can be seen from Fig. 6, the interaction indicated that Segment Orientation gave rise to systematic effects on Constant Error for the segments at larger distances from the interception axis. Post-hoc analysis of the interaction indicated that Constant Error was not affected by Segment Orientation for the 3.0 and 11.7-cm segment distances (all *p*s>.10). For these two segment distances Constant Error remained close to zero with linear regression slopes of +0.0013 cm/deg (*r*(23) = +.75, p<.001) and −0.0001 cm/deg (*r*(23) = −.02, *ns*), respectively. While the correlation between Constant Error and Segment Orientation was significant for the 3.0-cm segment distance, the slope of the relation indicated that this effect was negligible for the present purposes: a 45°-variation in Segment Orientation was associated with a change in Constant Error of less than 0.05 cm. For the 20.3 and 29.0-cm segment distances, however, Constant Error considerably varied with Segment Orientation, with the largest effect for the 29.0-cm segment distance (all *p*s<.05). Linear regression of Constant Error onto Segment Orientation demonstrated slopes of −0.0418 cm/deg (*r*(23) = −.98, p<.001) and −0.0661 cm/deg (*r*(23) = −.98, p<.001) for the 20.3-cm and 29.0-cm segment distances, respectively. For these latter distances the slopes were important, as a 45°-variation in Segment Orientation gave rise to changes in Constant Error in intersection locus estimates of 1.88 and 2.98 cm, respectively.

Overall, Experiment 2 reliably replicated the pattern of results reported by Arzamarski et al. [28]. The static line-intersection extrapolation task revealed errors in the estimated intersection locus that varied as a function of the distance from the axis of hand movement and the orientation of the segment presented. The systematic errors observed for the larger segment distances when the segment's orientation deviated from 0° (i.e., from perpendicular to the participant's movement axis) correspond to the perceptual biases referred to by Arzamarski et al. [28]. Thus, Experiment 2 validated our experimental setup for studying perceptual biases in the line-intersection extrapolation task.

## Experiment 3: Line-intersection extrapolation for bias-controlled trajectories

The goal of Experiment 3 was to test whether the systematic nature of the errors in estimated intersection locus observed in Experiment 2 could be used to control such perceptual biases. In other words, we sought to determine whether we could create a set of trajectories with zero perceptual bias with respect to the future ball arrival position at each point in each trajectory. To this end, we mathematically characterized the constant error (CE) in intersection locus estimates observed in Experiment 2 as a continuous function of Segment Distance (SegDist) and Segment Orientation (SO). For the two largest segment distances, we fitted the equation CE  =  k_1_ + k_2_*SO + k_3_*SegDist + k_4_*SO*SegDist + k_5_*SO^2^ + k_6_*SegDist^2^ + k_7_*SO^2^*SegDist + k_8_*SO*SegDist^2^. For the two closest segment distances, we considered CE to be equal to zero. The equation was not intended to provide any kind of generic model; it simply served to capture the effects observed as closely as possible. The fit accounted for 87.0% of the total variance, for a mean error of estimation of 0.06 cm. The resulting coefficients (CE in cm; SO in degrees) were 0.57346, −0.05680, 1.63140, 0.67885, 0.58676, 0.00134, 0.01601, and 0.00409 for k_1_ to k_8_, respectively.

**Figure 7:**
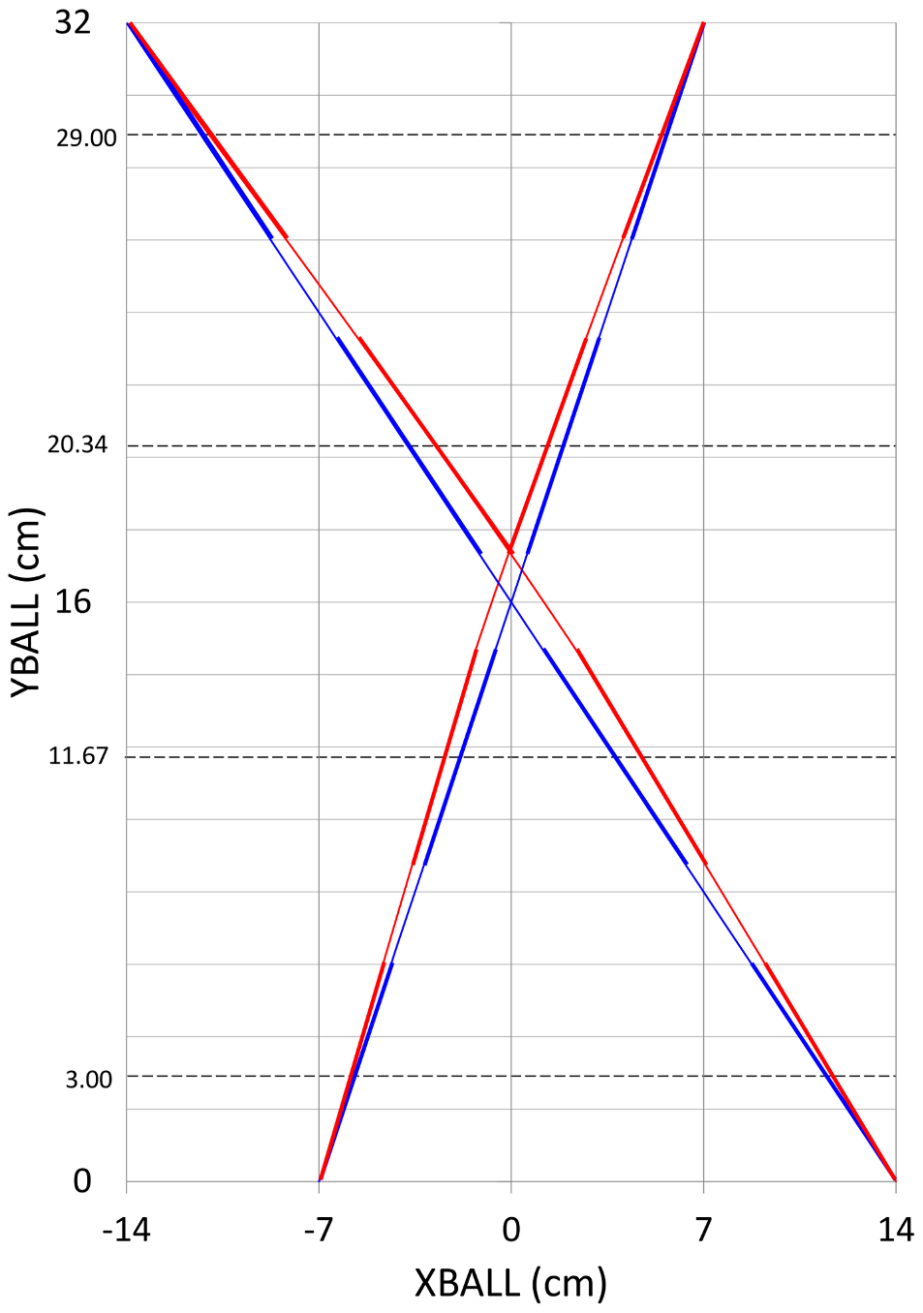
Examples of rectilinear and corresponding bias-controlled trajectories. Rectilinear trajectories for interception (Exp. 1, thin blue lines) and line segments for intersection-extrapolation (Exp. 2, thick blue line segments). Bias-controlled trajectories for interception (Exp. 4, thin red lines) and line segments for intersection-extrapolation (Exp. 3, thick red line segments). The exemplary trajectories shown are BDP = −14 cm to BAP = +14 cm and BDP = +7 cm to BAP = −7 cm.

Trajectories with a model-predicted zero bias with respect to the future ball arrival position at each point were constructed in the following way. The first point on each trajectory was, of course, the ball departure position. For this point we selected the segment orientation that corresponded to a real intersection position shifted away from the trajectory's ball arrival position by a distance equal to the perceptual bias predicted for this segment orientation at this distance from the axis of hand movement. By matching the shift in the real intersection location to the predicted error in its estimate, the predicted error with respect to the future ball arrival position was zero. Linear extrapolation of the bias-controlled trajectory orientation over a Y-distance of 0.1 cm provided the X-Y coordinates of the next point. The full trajectory was constructed by iterating the procedure. Examples of bias-controlled trajectories thus constructed are provided in Figure 7.

To test the validity of this bias-control logic, in Experiment 3 we again used the line-intersection extrapolation task. Our specific goal was to determine whether the newly constructed bias-controlled trajectories gave rise to (sufficiently precise) estimations of intersection locus that were no longer systematically biased.

### Materials and Methods

#### Participants

Fifteen right-handed participants (9 men and 6 women, mean age 21.7±1.8 yrs) voluntarily took part in the experiment. Six of them had participated in Experiment 2.

#### Task, Procedure, and Data Analysis

The experimental set-up was the same as in Experiment 2, with the exception of one characteristic. Instead of using segments from the rectilinear trajectories, the segments' position and orientation were extracted at the same four vertical distances as in Experiment 2 from the newly-created bias-controlled trajectories joining the same five ball departure positions to the same five ball arrival positions.

Participants performed 5 blocks of 100 trials, with the order of the 100 conditions (4 distances × 25 trajectories) randomized over trials in each block. No feedback was provided. Because segment orientations (SO) were adapted for each trajectory they could no longer be categorised into 9 levels, as was the case in Experiment 2. Therefore, as had already been done in Experiment 2, we evaluated the slope of the linear CE-SO relation for each segment distance.

### Results and Discussion

**Figure 8:**
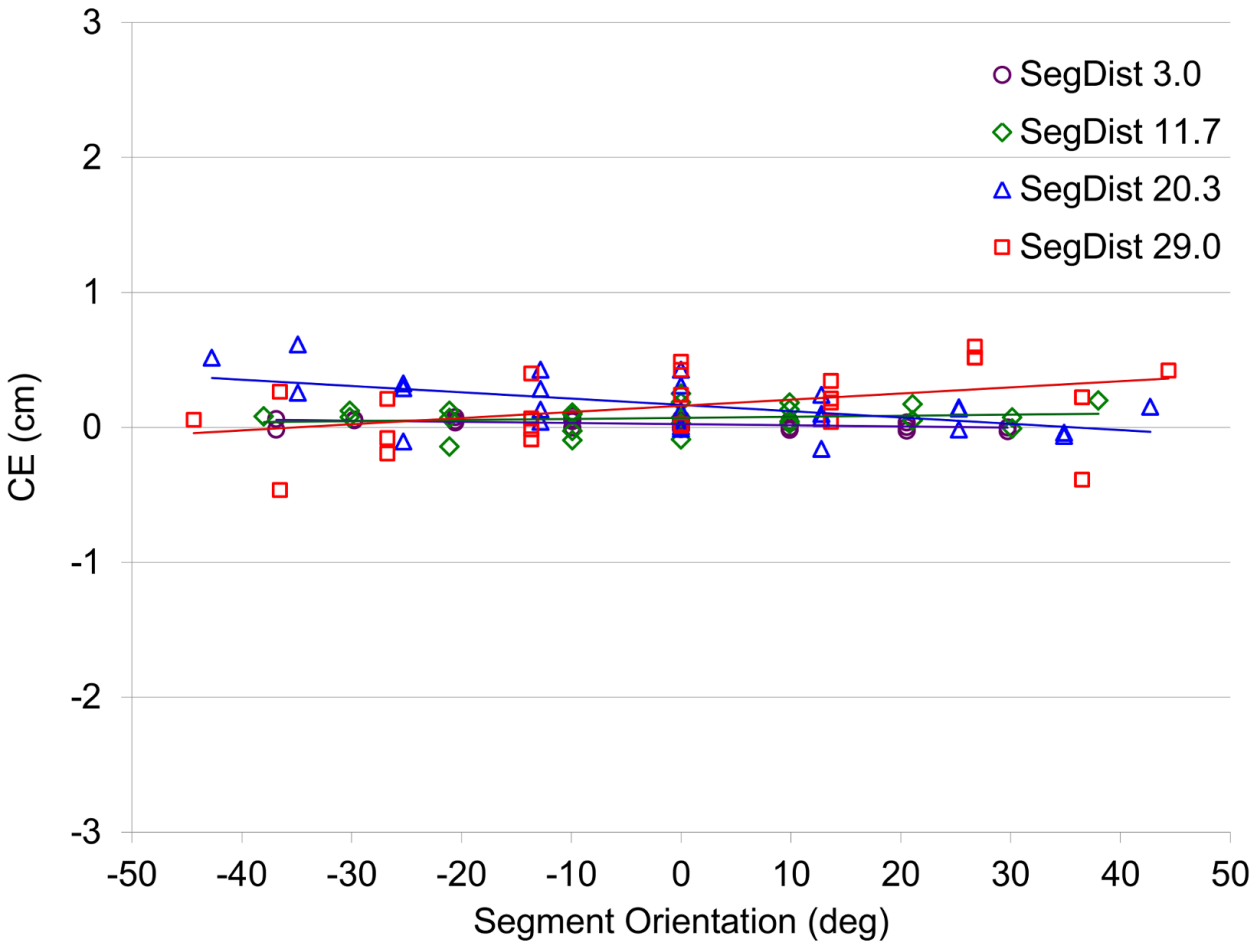
CE for intersection extrapolation as a function of segment orientation for the 4 segment distances (Exp. 3). CE: Constant Error. SegDist: Segment Distance.

As can be seen from Figure 8, the bias-control model allowed removing the systematic effect of segment orientation observed in Experiment 2. Constant Error in estimated intersection locus was less than 0.5 cm in all conditions. Linear regression slopes of the CE-SO relation were −0.0009, +0.0008, −0.0046, +0.0045 cm/deg for segment distances of 3.0, 11.7, 20.3, and 29.0 cm, respectively. Thus, notwithstanding the statistical significance of the CE-SO correlations for three of the four segment distances (*r*(23) = −.50, +.18, −.56, +.41, respectively), the variation in CE over a 45°-range of SO was always less than 0.2 cm. Such very small variations in CE were considered negligible for the present purposes. Another argument for neglecting such small slopes is the (difficult to explain) sign change of the (statistically significant) slopes for the 3.0-cm segment distance observed in Experiments 2 and 3.

Thus, the bias-control model allowed us to considerably reduce the pointing errors produced by the participants and, most importantly, to remove the systematic effects of Segment Orientation observed in Experiment 2 for the larger Segment Distances. We conclude that the bias-controlled trajectories are characterized by a (close to) zero perceptual bias at each point of each trajectory.

## Experiment 4: Intercepting balls moving along bias-controlled trajectories

Having established that the new set of trajectories effectively allowed controlling the perceptual bias, Experiment 4 tested whether the angle-of-approach effects observed in Experiment 1 would persist or vanish when intercepting balls moving along these bias-controlled trajectories. In the latter case, the angle-of-approach effect may indeed be ascribed to such biases [28]. In the former case, it cannot.

### Materials and Methods

#### Participants

Eight right-handed participants (5 men and 3 women, mean age 21.6±1.8 yrs) voluntarily took part in the experiment. All had participated in Experiment 3.

#### Task, Procedure, and Data Analysis

The task, procedure, and analyses of the data were identical to those of Experiment 1, the only difference residing in the characteristics of the ball trajectories used. The 25 rectilinear trajectories were replaced by the 25 new, slightly curved trajectories, linking the same five ball departure positions (Y = +32 cm; X = −14, −7, 0, +7, or +14 cm) and the same five ball arrival positions (Y = 0 cm; X = −14, −7, 0, +7, or +14 cm). Each of these 25 trajectories was constructed on the basis of the bias-compensation model developed on the basis of the results of Experiment 2 and tested in Experiment 3. Thus, the line-intersection extrapolation error was controlled to be close to zero at each point along all these trajectories.

### Results and Discussion

**Figure 9:**
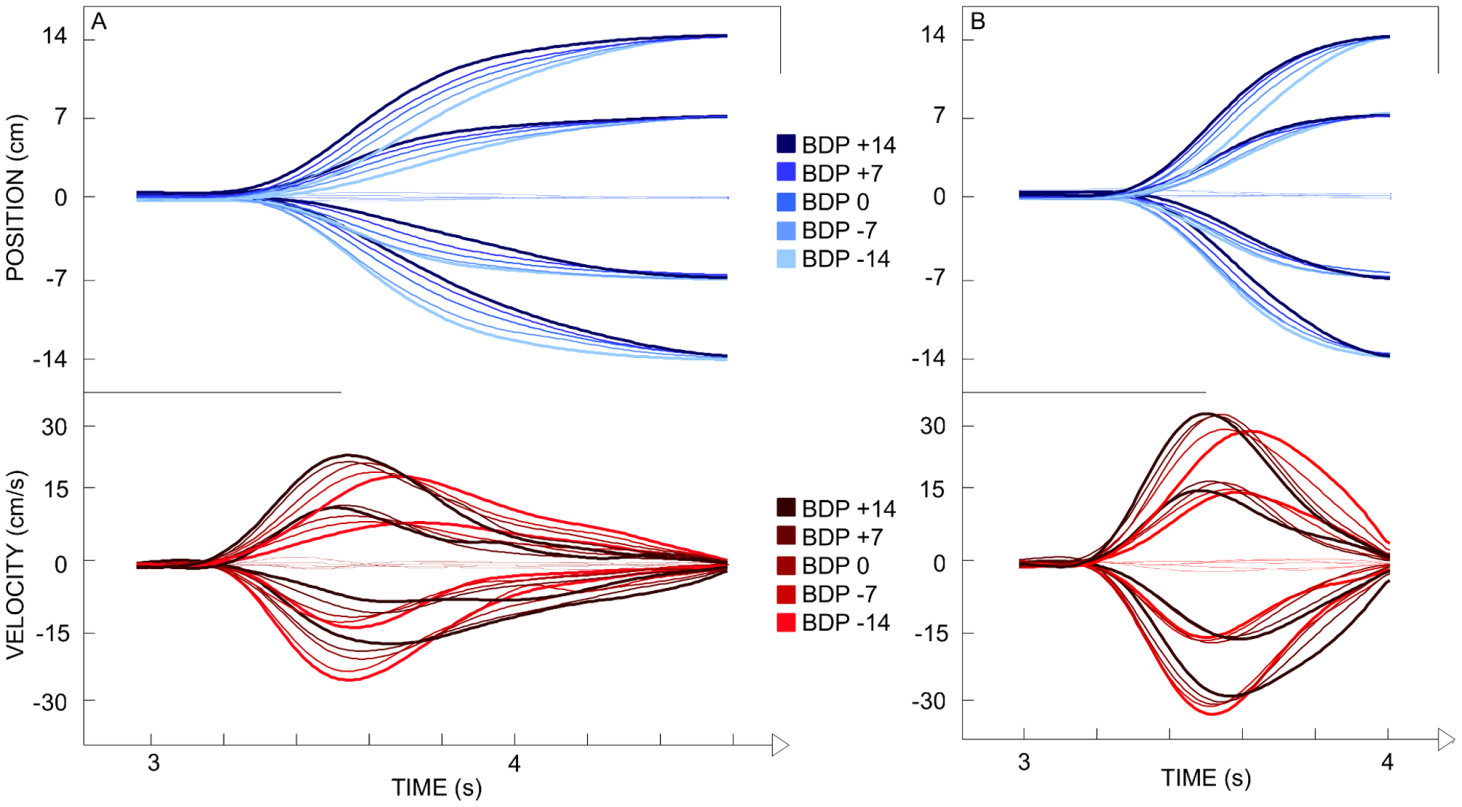
Ensemble averages of stylus position and velocity as a function of time (Exp. 4). The ball started moving 3(t_0_) on the screen. BDP: Ball Departure Position. Panel A: lower ball speed. Panel B: higher ball speed.

The ensemble averages of stylus position and velocity over time (Fig. 9) indicated systematic effects of ball departure position for all ball arrival positions (with the exception of X = 0 cm). These angle-of-approach effects were corroborated by the statistical analyses of kinematic characteristics of the movement patterns described below. Thus, the angle-of-approach effect observed in Experiment 1 continued to emerge even when participants intercepted ball moving along the bias-controlled trajectories.

#### Performance

Interception performance was again quite good, with 90.1% of the balls being intercepted. Overall, Constant Error was −0.02±0.64 cm. The ANOVA on Constant Error revealed a significant main effect of Ball Arrival Position (*F*(4, 28) = 4.46, *p*<.01) as well as a significant Ball Speed × Ball Arrival Position interaction (*F*(4, 28) = 4.00, *p*<.05). Post-hoc analysis of the interaction demonstrated that these effects were due to the higher Constant Error observed in the high ball speed condition for ball arrival position  = +14 cm. As can be seen from Fig. 9, these effects were again quite modest.

#### Movement kinematics

**Figure 10:**
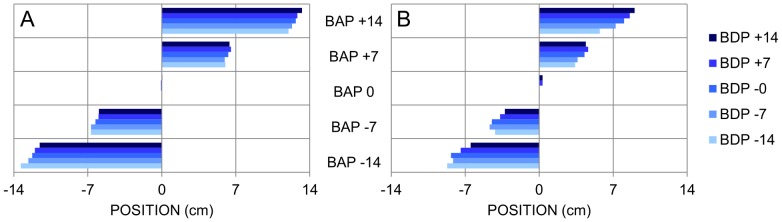
Pos-400 as a function of BDP and BAP (Exp. 4). Pos-400: Stylus position at 400 ms before the ball reached the interception axis. BDP: Ball Departure Position. BAP: Ball Arrival Position. Panel A: lower ball speed. Panel B: higher ball speed.

The ANOVA on Pos-400 revealed significant main effects of Ball Departure Position (F4, 28) = 57.80, p<.001) and Ball Arrival Position (*F*(4, 28) = 715.34, *p*<.001), as well as significant first-order interactions for Ball Speed × Ball Departure Position (*F*(4, 28) = 4.37, *p*<.01), Ball Speed × Ball Arrival Position (*F*(4, 28) = 119.61, *p*<.001) and Ball Departure Position × Ball Arrival Position (*F*(16, 112) = 8.61, *p*<.001). Finally, the second-order interaction Ball Speed × Ball Departure Position × Ball Arrival Position was also significant (*F*(16, 112) = 3.97, *p*<.001). Post-hoc analysis of the overarching interaction brought out the following points (see Fig. 10). Overall, Pos-400 was further away from the starting position for (a) balls moving at the lower speed (all *p*s<.05) and (b) balls moving towards farther arrival positions (all *p*s<.05). For each arrival position Pos-400 varied systematically with the ball's angle of approach (at least two significant Ball Departure Position comparisons at each Ball Arrival Position, except for Ball Arrival Position  = 0 cm). The smaller the (absolute) angle of approach, the closer the stylus was to the future ball arrival position. The effect of angle of approach was observed for both ball speeds.

**Figure 11:**
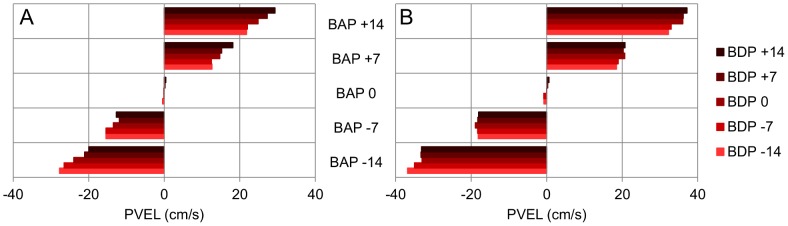
PVel as a function of BDP and BAP (Exp. 4). PVel: Peak Velocity. BDP: Ball Departure Position. BAP: Ball Arrival Position. Panel A: lower ball speed. Panel B: higher ball speed.

The ANOVA on Peak Velocity revealed significant main effects of Ball Departure Position (*F*(4, 28) = 26.24, *p*<.01) and Ball Arrival Position (*F*(4, 28) = 254.43, *p*<.001) as well as significant first-order interactions for Ball Speed × Ball Arrival Position (*F*(4, 28) = 100.95, *p*<.001) and Ball Departure Position × Ball Arrival Position (*F*(16, 112) = 3.79, *p*<.001). Post-hoc analysis of the interactions revealed the same effects (see Fig. 11) as those observed in Experiment 1. First, Peak Velocity was systematically larger when the distance between the starting position and the ball arrival position was larger (all *p*s<.05). Second, for each combination of ball departure position and ball arrival position Peak Velocity was systematically larger when the ball moved faster (all *p*s<.05). Finally, for each ball arrival position, Peak Velocity systematically varied with ball departure position (at least one significant Ball Departure Position comparison at each Ball Arrival Position, except for Ball Arrival Position  = 0 cm), with smaller Peak Velocities being attained for ball trajectories with larger (absolute) angles of approach. This angle-of-approach effect was observed for both ball speeds.

The angle-of-approach effects on the kinematics of interception movements observed in Experiment 1 for rectilinear ball trajectories were still present when participants intercepted balls moving along the bias-controlled trajectories. We therefore conclude that such angle-of-approach effects cannot be (fully) ascribed to perceptual biases in detecting XB_1_-based information.

## General Discussion

In the present series of experiments we addressed the control of lateral interception movements. In line with the literature [1], [24]–[28], the pattern of interceptive hand movement varied when participants intercepted balls converging onto the same interception location, arriving there after the same ball motion duration while following different trajectories. This persistent angle-of-approach effect militates against a predictive control strategy and provides compelling evidence in favour of the organization of movement on the basis of prospective control.

In prospective-control models of lateral interception [1], [2], [25], [27], [30], [31] the hand is continuously attracted toward an informationally-specified, time-evolving position along the interception axis. Controversy continues to exist with respect to the characteristics of the spatial information used: The two most prominent candidates are the current lateral ball position (XB_0_ [1], [2], [24], [25], [31]) and the ball's future crossing position if the current direction of ball motion is maintained (XB_1_ [27], [28], [30]). XB_1_ is invariant over rectilinear ball trajectories while XB_0_ is not (see Fig. 1). The finding of systematic angle-of-approach effects in a study of lateral interception using rectilinear ball trajectories led Montagne et al. [24] to conclude against the use of XB_1_-based information. However, Arzamarski et al. [28] argued that the observed angle-of-approach effect resulted from participants using perceptually-biased XB_1_-based information.

The perceptual-bias explanation proposed by Arzamarski et al. [28] was based on the inference that perceptual biases observed in a static line-intersection extrapolation task would generalize to the dynamic interception task. The present study was designed to test this perceptual-bias account of the angle-of-approach effect in lateral interception. To this end we developed a set of slightly curvilinear trajectories that effectively removed the perceptual biases that were held responsible for angle-of-approach effects by Arzamarski and colleagues. To generate ball motion along these bias-controlled trajectories we needed to rely on an interception task using virtual balls. Thus, the first step of the present study was to validate our experimental setup for the lateral interception of virtual balls moving across the screen of a large-sized interactive graphics tablet (see Fig. 2). This was achieved in Experiment 1 where participants intercepted balls moving along rectilinear trajectories from one of five departure positions on the top of the screen to one of five arrival positions at the bottom of the screen: We found clear angle-of-approach effects, replicating those known from previous studies [1], [24]–[28]. Next, we needed to validate the experimental setup for the line-intersection extrapolation task used by Arzamarski et al. [28] to determine perceptual biases. This was achieved in Experiment 2 where participants estimated the (extrapolated) intersection locus of static line segments on the lateral-interception line: We found estimation errors (i.e., perceptual biases) similar to the ones reported by Arzamarski et al. [28]. These first two experiments thus validated our experimental setup for the present purposes.

The next step was to design and validate a new set of bias-controlled trajectories. Using the systematic nature of the perceptual biases observed on the line-intersection extrapolation task of Experiment 2, we derived a set of slightly curvilinear trajectories for which the estimated intersection locus continuously coincided with the arrival positions of the original rectilinear ball trajectories (see Fig. 7). Experiment 3 demonstrated that our corrections for the perceptual biases had been successful: For line segments tangential to these trajectories participants' estimations of intersection locus was consistently close to the future ball arrival position. Thus, we were finally ready to test Arzarmarski et al.'s [28] perceptual-biases account for angle-of approach effects in lateral interception of balls traveling rectilinear trajectories. As in Experiment 1, in Experiment 4 we had our participants intercept balls moving from one of five departure positions to one of five arrival positions. Now, instead of rectilinear trajectories we used the bias-controlled trajectories. Our main finding was that the angle-of-approach effect persisted, also with trajectories that had been designed and tested to correct for perceptual biases. This result shows that perceptual biases are not responsible for the angle-of-approach effects reported here and in the literature [1], [24]–[28]. Because the perceptuomotor bias explanation [28] of the angle-of-approach effect does not hold under experimental scrutiny, the implication is that the on-line visual control of lateral interception is not based on information about XB_1_ exclusively [27], [28], [30]

We suggest that the present results indicate a combined use of information about XB_1_ and XB_0_. That is to say, although a control based only on information about XB_1_ was ruled out by the current study, an account based completely on information about XB_0_ also seems untenable. Prospective control models based on information about XB_0_ [1], [2], [31] not only predict angle-of-approach effects. They also predict that balls that will eventually arrive at the hand's starting position should give rise to an initial hand movement away from the starting position, in the direction of the time-evolving XB_0_ (see Fig. 1). As XB_0_ approaches the future interception location during the course of ball motion, hand movement should be reversed, with the ball finally being intercepted at the hand's initial position. Although Montagne et al. [24] indeed reported such movement reversals, later studies, including the present, have not replicated these. At present we have no explanation for this discrepancy. The robust finding of small but systematic angle-of-approach effects is nevertheless compatible with the use of XB_0_-based information. However, the absence of systematic reversal movements militates against an exclusive use of XB_0_-based information. Thus, the control of lateral interception appears to be based on an informational quantity relating to both XB_0_ and XB_1_. Interestingly, for participants placed in the role of a goalkeeper in football such a composite informational quantity was recently found to explain their judgements of whether a ball would enter the goalmouth or not [35]. Models of prospective control based on such a composite informational variable need to be developed and their predictions tested in new experiments.
